# Oxidative Stress and Assisted Reproduction: A Comprehensive Review of Its Pathophysiological Role and Strategies for Optimizing Embryo Culture Environment

**DOI:** 10.3390/antiox11030477

**Published:** 2022-02-28

**Authors:** Ashok Agarwal, Israel Maldonado Rosas, Christina Anagnostopoulou, Rossella Cannarella, Florence Boitrelle, Lina Villar Munoz, Renata Finelli, Damayanthi Durairajanayagam, Ralf Henkel, Ramadan Saleh

**Affiliations:** 1 American Center for Reproductive Medicine, Cleveland Clinic, Cleveland, OH 44195, USA; finellirenata@gmail.com (R.F.); rhenkel@uwc.ac.za (R.H.); 2 Citmer Reproductive Medicine, IVF LAB, Mexico City 11520, Mexico; imaldonado@citmer.mx (I.M.R.); lvillar@citmer.mx (L.V.M.); 3 Unit of Reproductive Medicine—Embryo ART, Lito Maternity Hospital, 11524 Athens, Greece; anagnostc@gmail.com; 4 Department of Clinical and Experimental Medicine, University of Catania, 95123 Catania, Italy; rossella.cannarella@phd.unict.it; 5 Department of Biochemistry and Molecular Biotechnology, University of Massachusetts Medical School, Worcester, MA 01605, USA; 6 Reproductive Biology, Fertility Preservation, Andrology, CECOS, Poissy Hospital, 78300 Poissy, France; florenceboitrelle@yahoo.fr; 7 Department BREED, UVSQ, INRAE, Paris Saclay University, 78350 Jouy-en-Josas, France; 8 Faculty of Medicine, Universiti Teknologi MARA (UiTM), Sungai Buloh Campus, Jalan Hospital, Sungai Buloh 47000, Selangor, Malaysia; damayanthi.d@gmail.com; 9 Department of Metabolism, Digestion and Reproduction, Imperial College London, London W2 1NY, UK; 10 Department of Medical Bioscience, University of the Western Cape, Bellville, Cape Town 7530, South Africa; 11 LogixX Pharma, Theale RG7 4AB, UK; 12 Department of Dermatology, Venereology and Andrology, Faculty of Medicine, Sohag University, Sohag 82524, Egypt; salehr2010@yahoo.com; 13 Ajyal IVF Center, Ajyal Hospital, Sohag 82524, Egypt

**Keywords:** antioxidants, culture media, embryo development, oxidative stress, IVF, in vitro fertilization

## Abstract

Oxidative stress (OS) due to an imbalance between reactive oxygen species (ROS) and antioxidants has been established as an important factor that can negatively affect the outcomes of assisted reproductive techniques (ARTs). Excess ROS exert their pathological effects through damage to cellular lipids, organelles, and DNA, alteration of enzymatic function, and apoptosis. ROS can be produced intracellularly, from immature sperm, oocytes, and embryos. Additionally, several external factors may induce high ROS production in the ART setup, including atmospheric oxygen, CO_2_ incubators, consumables, visible light, temperature, humidity, volatile organic compounds, and culture media additives. Pathological amounts of ROS can also be generated during the cryopreservation-thawing process of gametes or embryos. Generally, these factors can act at any stage during ART, from gamete preparation to embryo development, till the blastocyst stage. In this review, we discuss the in vitro conditions and environmental factors responsible for the induction of OS in an ART setting. In addition, we describe the effects of OS on gametes and embryos. Furthermore, we highlight strategies to ameliorate the impact of OS during the whole human embryo culture period, from gametes to blastocyst stage.

## 1. Introduction

Assisted reproductive techniques (ARTs) represent an important treatment option for infertile couples and its utilization has constantly increased with growth rates between 2.4% and 18.3% per annum in Europe, USA, Australia, and New Zealand in the years from 2012 to 2016 [1], resulting in about 1.3 million ART cycles in 2016 in these areas. Such an increase may be caused by several factors, including socio-economic [2] and environmental factors [3]. This is also paralleled with the observation of the temporal decline of human fertility in many countries [4]. Despite great advances in the field, the success rate of ART procedures remains unsatisfactory in many cases [5] and requires further improvements.

Oxidative stress (OS) has been established as an important factor that can negatively affect ART outcomes [6,7,8,9]. Oxidative stress is defined as an imbalance between the reactive oxygen species (ROS) and the total amount of antioxidants (AOXs) in favor of the oxidants [10]. At low concentrations, ROS act physiologically as signaling molecules in several processes [11,12]. In male reproduction, these redox mechanisms play an important role in the regulation of numerous functions, including spermatogenesis, chromatin condensation, sperm maturation during transport in the epididymis, sperm hyperactivation, capacitation, acrosome reaction, and sperm-oocyte interactions [13,14,15]. In female reproduction, redox homeostasis is reported to be critical for folliculogenesis, implantation, and placentation [15].

On the other hand, abnormally high levels of ROS can damage cellular lipids, organelles, and DNA, alter enzymatic function, and trigger apoptosis [11,16]. ROS-induced lipid peroxidation leads to the production of highly reactive and mutagenic products, such as malondialdehyde (MDA), an important indirect molecular marker of OS [17,18]. Sperm are particularly susceptible to OS due to the high content of omega-3 polyunsaturated fatty acids (PUFA) in sperm cell membranes [19], and limited AOX defenses in their cytoplasm [20]. Lipid peroxidation of sperm causes mitochondrial membrane damage and dysfunction, and reduced adenosine triphosphate (ATP) production [21]. Additionally, lipid peroxidation reduces the fluidity of the sperm membrane, thus interfering with capacitation, acrosome reaction, and sperm-oocyte fusion [20,21]. Furthermore, pathological levels of ROS can cause sperm DNA fragmentation (SDF), either directly or indirectly via MDA [17,18]. Three recent meta-analyses revealed a correlation between elevated SDF and reduced pregnancy rates following intrauterine insemination (IUI) [22] and in vitro fertilization (IVF) [23,24,25], although no significant association was reported following intracytoplasmic sperm injection (ICSI) [23,24,25]. Moreover, increased SDF has been correlated with low embryo quality [23], high miscarriage rates [23,26], and low live birth rates [27] after IVF and ICSI. In females, high levels of ROS have been observed in several pathological conditions, such as polycystic ovary syndrome, endometriosis, spontaneous abortions, preeclampsia, and intrauterine growth retardation [28].

**Figure 1:**
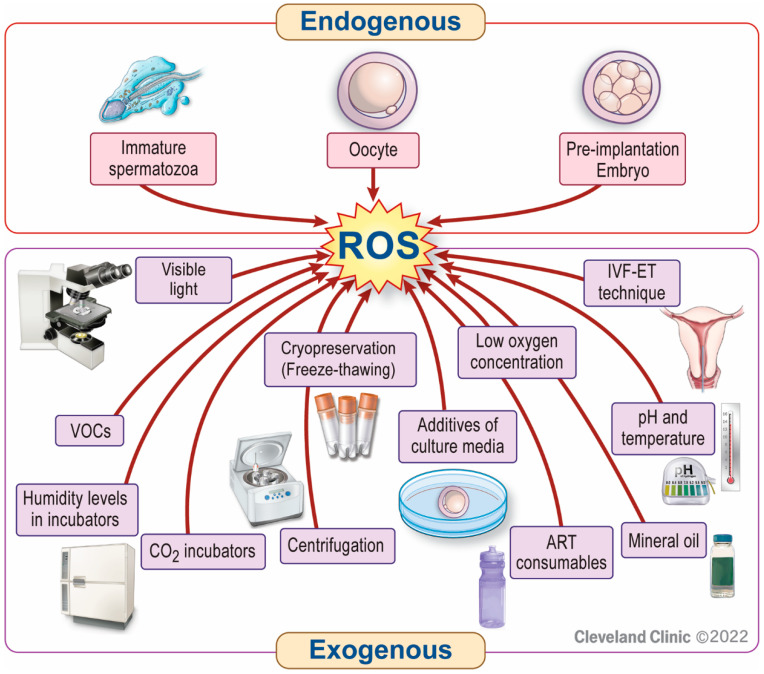
Factors responsible for increased ROS generation in an ART setting. Reactive oxygen species (ROS) can be produced intracellularly from immature sperm, oocytes, or embryos. External sources or triggers of ROS production include inappropriately high or ultra-low oxygen tension, contamination of laboratory air, CO_2_ incubators, or ART consumables (e.g., plastics, bisphenols) with volatile organic compounds (VOCs). In addition, centrifugation, visible light, temperature, humidity levels in incubators, mineral oil, additives of culture media, the in vitro fertilization (IVF)-embryo transfer (ET) technique, and cryopreservation of gametes or embryos also contribute to ROS generation in an ART setting.

In an ART setting, several factors can be responsible for increased ROS generation, leading to suboptimal ART outcomes [29] (Figure 1). ROS can be produced intracellularly, from immature sperm, oocytes, and embryos. The latter may be particularly vulnerable to OS due to a lack of the protective antioxidants in their physiological microenvironment during in vitro ART conditions [30]. In addition, several external factors may induce OS in an ART setup, including atmospheric oxygen (pO_2_, 20–21%) [31,32,33,34,35]. Most body tissues, including the fallopian tubes [36], function properly at oxygen concentrations of 4% to 10% [37,38]. The toxic effect of atmospheric oxygen levels on embryos has already been shown by Pabon et al. (1989) [39] and Umaoka et al. (1992) [40]. This effect is mediated by excessive production of ROS causing OS [41]. On the other hand, a really low oxygen tension (<2%) is also detrimental to embryonic development due to: (i) inhibition of essential developmental steps [42] and (ii) release of excess mitochondrial ROS [43]. The laboratory air, the gases used, ART consumables, and the quality of culture media can also contribute to OS in an ART setting [44,45,46,47]. In addition, visible light, temperature, and humidity can directly or indirectly trigger OS [46,48]. Furthermore, ROS can be generated during the cryopreservation-thawing process of gametes or embryos, thus increasing the risk of ROS-induced cryo-damage [49,50]. All these factors can act at any stage during ART, from gamete preparation and fertilization to embryo development until the blastocyst stage. Hence, strategies to reduce the risk of OS in ART should include optimization of the laboratory environment, sperm preparation techniques, embryo culture media, and cryopreservation protocols [29].

The objectives of this review are to: (1) discuss the in vitro conditions responsible for the induction of OS in an ART setting, (2) describe the effects of OS on gametes and embryos, and (3) highlight strategies to minimize the impact of OS during the entire human embryo culture period, from gametes to blastocyst stage.

## 2. Mechanism(s) of OS Production in ART

### 2.1. Stages of OS Generation during ART

#### 2.1.1. Handling of Gametes

In an ART setting, gametes undergo in vitro manipulation, which inevitably expose these cells to OS [51]. This is particularly true in sperm that have a limited amount of intracellular AOXs [52]. During sperm preparation for ART, semen samples are usually washed, and the seminal plasma removed [51]. Seminal plasma physiologically provides AOX protection, as it contains a wide range of both enzymatic [e.g., superoxide dismutase (SOD), catalase, glutathione peroxidase (GPx), etc.] and non-enzymatic (e.g., vitamin C, vitamin E, glutathione, etc.) compounds with antioxidant properties [52]. In addition, during swim-up, sperm may come in close contact with leukocytes and immature germ cells (both of which represent major sources of ROS) before migrating to the surface [51]. Furthermore, the centrifugation steps of sperm preparation techniques further result in increased ROS generation [52], with longer centrifugation time being associated with higher amounts of ROS and sperm damage [53]. 

In addition, after oocyte retrieval, cumulus cells comprising the cumulus oocyte complex are removed. This should be performed as soon as possible under controlled temperature, as culture dishes can lose temperature quickly during oocyte preparation under the laminar flow hood [54] and during pipetting [55]. Temperature changes in buffered media can make pH conditions unstable, with detrimental consequences on oocytes [56,57]. Despite the improvement in culture medium composition, the natural environment in the female reproductive tract cannot be faithfully reproduced in vitro.

#### 2.1.2. Embryo Cryopreservation

Currently, the use of frozen embryos in ART programs has considerably increased compared to the past. This is attributed to the development of the vitrification method and the adoption of the freeze-all strategy, as well as the possibility to obtain a surplus of embryos and avoid hyperstimulation syndrome [58]. Before the development of vitrification, slow-freezing was the most widely used method for gamete and embryo cryopreservation. However, slow-freezing has been shown to increase the formation of intra- and extra-cellular ice crystals, thus inducing cellular damage, apoptosis [59,60], and increased OS. Accordingly, several conditions potentially leading to OS arise during cryopreservation. These include dilution of sperm samples (and seminal fluid), exposure to toxic cryoprotectants, and pH and osmotic changes induced by cooling to cryogenic temperatures and rewarming for thawing. In line with this, a significantly increased ROS production has been reported in human gametes, as well as in mice and human embryos, during cryopreservation [61,62,63,64]. In contrast, the formation of ice crystals is lower when vitrification is performed, with lower osmotic shock due to ultra-rapid cooling [65]. Accordingly, vitrification has been associated with higher oocytes and embryos survival, and improved metabolism and blastocyst formation rate compared to slow-freezing [66,67]. Furthermore, a meta-analysis of 6590 cycles reported that embryo vitrification has no detrimental impact on ART outcomes in terms of implantation, pregnancy, and live birth rates [65]. 

### 2.2. Role of Laboratory Factors in OS Generation during ART

In vitro embryo culture, as a part of ART treatment, is a complex system that intends to mimic in vivo conditions. A number of factors can affect its success, as discussed below.

(a) Oxygen

ROS, such as hydroxyl radicals (^•^OH), superoxide anion (O_2_^•−^), or hydrogen peroxide (H_2_O_2_), are extremely reactive with very short half-life times in the nanosecond (10^−9^ s) (^•^OH; hydroxyl radicals) to millisecond range (10^−3^ s) (O_2_^•−^; superoxide radicals) [68]. Thus, every organism must adapt to this oxidative environment. In eukaryote cells, the mitochondria are not only consuming oxygen for the energy and ATP production, but it has been shown that in the course of the electron transfer process, about 1–5% of the consumed oxygen is converted into ROS [69]. A certain limited amount of ROS is crucial for the induction of essential physiological processes such as sperm capacitation [70] or the modulation of transcription factors such as mitogen-activated protein kinases [71]. Moreover, redox homeostasis plays a vital role in embryogenesis, embryo development, and the onset of pregnancy [42].

In the female reproductive tract, physiological amounts of ROS are important for normal follicle development, oocyte maturation, ovarian steroidogenesis, and luteolysis [72,73]. During early implantation, the embryo is challenged with almost anoxic conditions in the uterus [36]. This change in the oxygen tension from about 2% to 5% in the ovaries to almost anoxic is accompanied by a change in the embryonic metabolism from oxidative phosphorylation with low glucose levels in the fallopian tube to glycolysis with high glucose levels in the uterus at implantation and early post-implantation [42], favoring preimplantation embryo development and placentation [74]. After placentation, during the so-called post-implantation embryonic metabolism switch, the glycolysis rate decreases again, and oxidative phosphorylation increases [75]. Then, the placental blood circulation is established and the oxygen levels increase triggering proliferation, cell growth, and differentiation [76]. This indicates that a finely controlled redox balance is required for fertilization, embryo development, implantation, and further differentiation [77]. 

Studies with human embryonic stem cells suggested that physiological oxygen concentration benefits embryos by reducing cellular stress and preventing epigenetic alteration and irreversible X chromosome inactivation [78,79]. Optimum oxygen concentration for culturing mammalian embryos in vitro has been widely debated by the scientific community over the past few decades. Fertilization, cleavage, pregnancy, and implantation rates were not significantly different when oxygen concentrations of 5% or 20% were used during the culture of embryos up to day 2 or day 3 [80]. However, a Cochrane systematic review indicated that embryo culture at low oxygen concentration of 5% has a clinical benefit on ART outcome compared with atmospheric concentrations [81]. The practice guidelines of the European Society of Human Reproduction and Embryology (ESHRE) recommended culture of human embryo at low oxygen concentrations ranging from 2% to 8% [82]. More recent studies indicated that low oxygen levels (5%) during embryo culture favor increased the number of mitochondria in blastomeres in mice [83], and blastocyst development and pregnancy rates [84,85,86] in humans as compared to culture in higher (20%) oxygen concentration. Embryos are particularly sensitive to OS induced by high (20%) atmospheric oxygen concentration at the early stages before embryonic genome activation [87]. ROS levels in media cultured under 5% oxygen were found to be lower than that under atmospheric oxygen concentrations [87].

A study by Kaser et al. [88] reported that embryos cultured at ultra-low oxygen concentrations (2%) are more likely to develop into good blastocysts than the respective sibling embryos cultured under 5% oxygen. On the other hand, a recent study showed that lowering oxygen tension from 5% to 2% from day 3 onwards seems not to further improve embryo development during extended human embryo culture [89].

(b) Volatile organic compounds

Volatile organic compounds (VOCs) are mainly man-made chemicals that can vaporize under normal atmospheric conditions and have an odor such as benzene, toluene, formaldehyde, and ethanol [44]. Contrary to common belief, the IVF lab may be less clean in terms of VOCs compared to outside air that is relatively VOC free [44]. Common sources of VOCs are construction materials, wood furniture, paints, adhesives, motor vehicles emissions, and cleaning products [90]. Additionally, they may originate from various sources in the IVF laboratory such as microorganisms, transferred from the overlying oil to the culture medium, tanks of medical gases used for embryo culture, plastic petri dishes, or plastic packages of disposable supplies [91]. Several studies have documented the detrimental effect of VOCs on human and animal cells in vitro and have linked OS induction with the toxicity of several pollutants [92,93,94,95]. ART outcomes (pregnancy and live birth rates) are indeed linked to air quality of the ART laboratory [90,91]. In humans, environmental pollution has been correlated with high levels of VOCs in human semen, which also corresponded to poorer semen quality in the young males who were inhabiting the area [96]. However, the role of VOCs in the pathogenesis of OS in an ART setting is not clear and warrants further research.

(c) pH

- The pH of embryo culture media

One of the most crucial culture parameters is the external pH (pHe) of the culture medium. The working pHe in bicarbonate buffered culture media is only achieved after equilibration in a 37 °C incubator under a CO_2_ gas phase dissolved in the solution [97]. Denuded oocytes have no or limited ability to maintain internal pH (pHi) at 7.1 [57,98]. Therefore, the pHe needs to be calibrated at a slightly higher pHi between 7.2 and 7.3 [98,99,100]. 

Embryos at the morula and blastocyst stages are more likely to stabilize their pHi than early cleavage-stage embryos and oocytes [101]. This can be achieved by extruding the excess of protons out of the cells or buffering their cytosol by the import of bicarbonate through specific transport systems present in the cell membrane [100]. If a non-oil covered culture dish is removed from the incubator, and subjected to a non-gassed atmosphere, the pH will increase in less than one minute [97]. In the latter study, the authors indicated that if the dish containing embryos is removed from the incubator, but kept at 37 °C, the pH begins to rise immediately, and reaches a maximum pH of 7.77 (0.5 mL of medium in the well) or 7.52 (1.0 mL of medium in the well) after 5 min in room air. Other authors have compared the pH in dishes containing 5 mL of oil-free medium to those in drops of 50 μL media under oil, after exposure to ambient air outside the incubator [102]. In both cases, the pH became higher than 7.45 in two minutes. Long but small changes in temperature or CO_2_ concentration during embryo incubation strongly affect the culture media pH, impacting negatively on embryo development and clinical outcomes due to increased OS [54].

- The pH in handling media

The pH of human oocytes and embryos slightly varies during development, usually accepted as between 6.98 and 7.03 in oocytes [103] and 7.12 in embryos [97]. However, it is still unclear whether a pH of 7.2 is better or worse for human embryos than a pH of 7.3 in handling media [104]. Human oocytes and cleavage embryos are more able to regulate alkalinity in the cytosol induced by high levels of pHe but are unable to buffer acidosis. This suggests that the environment of external oocyte and cleavage embryos are not more acidic than that of the cell cytoplasm [105]. 

The increasing production of both ROS [106] and nitric oxide (NO) [107] in cells might be determined by changes on the gradient of pH in the mitochondria [106]. Thus, pH has been considered as another OS stimulator, specifically in the cellular mitochondrial respiratory chain [108].

(d) Temperature

In ART laboratories, the temperature of CO_2_ incubators has traditionally been adjusted to 37 °C to mimic in vivo conditions. However, reliance on the accuracy of temperature solely on the external display of the incubators is considered incorrect. For example, the internal temperature of bench top incubators may be 37 °C, while the display shows a higher temperature [109]. Large incubators also show internal temperature variations of around ±0.3 °C [110]. Since temperature of the incubators and heating plates is almost never a reflection of the temperature of the media, temperatures should be measured directly in the embryo culture medium during an ART-like procedure. These controls are to be done regularly with metrologically qualified thermometers. Despite several studies comparing culture at 37 °C versus 36 °C, there is currently insufficient data to suggest that incubation at a more physiological, cooler temperature improves pregnancy rates [111,112]. Embryonic cells tolerate lower temperatures better than higher temperatures, thus resulting in better pregnancy rates when the incubator environment is lower than 37 °C as opposed to >37 °C [113]. On the other hand, elevated temperatures may cause heat stress in embryos and increase ROS [114]. However, the ideal temperature for in vitro culture still remains an area of debate [54].

In order to stabilize the temperature during handling, it is important to consider how manipulations and environmental conditions can affect the heat loss of the culture media [115]. Oocyte cooling to 25 °C can temporarily disrupt the microtubules and microtubule organizing centers and displace the chromosomes, although the development to the blastocyst stage of those fertilized oocytes remains similar to non-cooled controls [116]. Similar results were obtained by exposing both human oocytes to room temperature during vitrification and thawing procedures, reporting clinical outcomes that were comparable to that of non-cooled cells [117]. It appears that many of the changes induced by low temperature are reversible in many but not all oocytes on their restoration to 37 °C for 1 h [118].

(e) Humidity

Humidification of incubators can affect the osmolarity of the embryo culture, which is considered of pivotal importance for ART success [119]. In fact, hypertonic media can heavily affect embryo development and clinical outcomes [119]. These detrimental effects are believed to occur by triggering several mechanisms, such as OS, cell shrinkage, DNA, or mitochondrial damage, cell cycle arrest, and apoptosis [120]. In line with this, a recent study compared the human ART outcomes of humid culture conditions (285–290 mOsm) with that of dry culture ones (308 mOsm), reporting a significantly higher rate of miscarriage in the former [120]. However, the last Cochrane systematic review reported no difference [121]. Culture in a dry incubator may exert negative effects on embryo development due to increased OS levels [119,120,121,122].

(f) Embryo culture media

The contribution of the culture medium to ROS production and OS of gametes during ART is not clear. A study indicated significantly higher ROS levels in different cell-free culture media compared to ROS levels in follicular fluids obtained from infertile women [123]. According to the latter study, the more complex the culture media, the higher the ROS levels that are present. In another study, the rates of good quality blastocyst formation were significantly higher when culture media with lower ROS levels were used [124].

Ammonia is produced by metabolic processes in cells and causes several deleterious damages due to its high toxicity. Ammonia is the major product from glutamine [125], and induces ROS overproduction [126] and decreases mitochondrial membrane potential (MMP) [127]. To reduce the major source of ammonia in embryo culture media, the unstable L-glutamine is replaced by alanyl-glutamine or glycyl-glutamine [128]. Apart from amino acids, other factors, such as the protein supplementation, might contribute to the ammonium accumulation in culture media that also show an ammonium buildup during storage at 2–8 °C and during incubation [129].

- Culture media renewal

IVF culture media have evolved from a stage-specific medium to a single medium to satisfy embryo needs by simulating its transportation in the oviduct. Renewal of the embryo culture medium diminishes excess ammonia [130]. The benefits of single-embryo culture over sequential medium culture include the reduction in the chances of embryonic loss, contamination, and handling errors, less embryo stress, and reduced temperature and pH changes, with no difference in embryo morphokinetics and development [130,131]. However, a study found no apparent advantage in using a single medium over that of sequential medium [132].

(g) Mineral oil

Mineral oil has been a standard component of embryo culture since the early 1960s [133], however, relatively little data exists on its potential impact on embryo development. Oil is a petroleum product that can harbor embryo toxins, including zinc and peroxides [134]. Oil is widely used during embryo culture as it prevents evaporation, and helps maintain the pH, temperature, and osmolarity of the culture medium [135].

Despite high quality control measures and rigorous testing of mineral oils, there have been reports of inadequate transport and storage conditions that result in detrimental effects on embryo development. Both oil storage time and high temperature storage can increase mineral oil peroxidation [136], with an increase in the peroxide value (POV), regardless of the oil used. This has deleterious effects on the embryo culture medium and the embryo [136,137]. Storage at room temperature in the dark for 2 months would not increase the POV, but longer storage of 6 to 15 months would increase it by 0.12 mEq/kg and 0.21 mEq/kg, respectively [137]. The POV could even reach very high values (in the order of 3.0 mEq/kg) after sun exposure for 7 weeks or UV exposure for 120 h. At elevated temperature (50 °C) in the dark, the POV increased by 0.04 and 0.10 mEq/kg in 20 and 50 days, respectively. Ideal oil storage conditions would be in the dark between 2 and 8 °C for some manufacturers or in the dark at room temperature for others [137]. 

Mouse embryo development up to the blastocyst stage is dependent on the POV value, with embryo death observed at day 3 for POV values of about 0.5 mEq/kg and at day 2 for POV values of 1 mEq/kg [137]. Therefore, it is important that the transport and storage conditions, as well as storage time is known and optimized. Some authors proposed an extended mouse embryo protocol to help detect poor quality oils [138]. Others recommended washing the oil using different washing media, such as distilled water, to decrease the POV and improve the oil quality [133,139]. 

Mineral oil may alter the embryonic culture conditions by interfering with osmolality regulation [135]. Some mineral oils can increase the osmolality of the culture medium with negative impact on embryo development [140]. The volume of the oil has recently been shown to play a role in increasing the osmolality of the embryo culture medium [119]. For example, if a microdroplet of culture medium is covered with 1300 µL of oil, evaporation is significantly decreased with subsequent reduction of osmolality, compared to using an oil volume of 700 µL. However, the low oil volume is not the only factor involved in increasing the osmolality of the embryo culture medium. Additional factors that may contribute to the rise in osmolality include the surface area-to-volume ratio of the culture medium, oil density, and oil thickness above the medium [141]. A study by Sifer et al. [142] showed significant differences in embryo quality depending on the type of oil used. However, more recently, a comparison of paraffin oil and mineral oil showed no differences in fertilization rate or live-birth rate after normal exposure during embryo culture to either oil [143]. In a very recent study, 13 marketed oils were tested [144]. The POV was very low for all the oils tested. The POV increased for all these oils after two weeks of exposure to sunlight. Even more disturbing is the fact that 2 oils out of the 13 tested and currently marketed were described as toxic to the embryo, after performing MEA tests. Thus, the choice of oil is important for the ART laboratory, to optimize the embryo culture. Well-designed, large-scale studies are warranted to address the role of oil in ART outcomes.

(h) Light

Light is proven to be harmful to embryo culture either directly or indirectly by causing oil peroxidation and photo-oxidation of culture medium [145,146]. The toxic effect of visible light on hamster embryos may be due to increased generation of ROS [48]. Such an observation was further confirmed in human cells [147]. In fact, specific wavelengths are reported to be absorbed by enzymes of the electron transport chain [148], resulting in increased generation of ROS. This impact is reduced by using specific filters, which are able to exclude radiation in the blue spectra [149]. It is still unclear how long a cell should be exposed to light before OS increases: a transient exposure as well as 5 min have been speculated [46,48].

(i) IVF consumables

Polystyrene- and polypropylene-based plastics routinely used in ART laboratories include centrifuge and oocyte collection tubes, pipette and filter tips, petri dishes, cell culture plates, culture medium bottles, and cryotube vials [150], as well as handling pipettes, syringes, and catheters. These consumables remain in close contact with the gametes or embryos for various durations and thereby could potentially exert a negative effect on them. Bisphenol A (BPA) is a plasticizer commonly used in polycarbonate plastics, and an endocrine disruptor with weak estrogenic activity. It has adverse effects on both the male and female reproductive system including epigenetic alterations during gamete development [151,152,153]. An earlier study reported that under regular conditions, plastic consumables do not leach BPA into the culture medium [154]. However, a more recent study detected Bisphenol-S (BPS), an analog of bisphenol-A, at nanomolar concentrations in culture media, probably originating from the medium’s plastic bottle or from the medium’s production process [150]. Despite BPS being generally less estrogenic and antiandrogenic compared to BPA [155], evidence from animal studies showed that low doses of BPS was sufficient to affect oocyte quality in vitro and induce OS [150,156], while low doses of another analog, Bisphenol AF (BPAF) disrupted oocyte maturation in vitro [157]. Additionally, plastic packages containing consumables may emit VOCs that have a detrimental effect on embryo development and implantation [45]. Therefore, off-gassing consumables for more than 24 h in the laminar flow hood allows substances, such as aldehydes, benzene, and styrene to dissipate, and is a common practice in many IVF laboratories [158].

## 3. Gamete and Embryo Responses to OS in ART

- Embryo plasticity

The developmental plasticity of the early embryo is an impressive feature [159]. Single blastomeres from 2-cell and 4-cell mouse embryos are shown to be totipotent, being capable of producing a fully formed individual, while morula and early blastocyst cells are considered pluripotent [160]. As a result, loss of one or more blastomeres due to the freeze-thaw procedure or biopsy technique in day 3 may be compensated, and not affect embryo survival and implantation [161,162]. Additionally, features such as direct cleavage, irregular division, multinucleation, and mosaicism (considered a sign of a non-viable or non-transferable embryo) have been shown to be manageable and reversible in some cases, based on data from preimplantation genetic testing [163,164,165]. Progress in time-lapse technology and artificial intelligence image analysis has allowed for the study and reporting of the reorganization of fragments, exclusion of blastomeres or fragments, probably used by the embryo as a means to self-correct [166,167].

However, although preimplantation embryos can adapt to adverse environmental conditions and grow to blastocysts and implant, cellular stress may result in permanent and inheritable epigenetic effects [168]. Adaptation to environmental challenges may result in the development of different phenotypes through epigenetic regulation that can persist for more than one generation [169]. Interestingly, no significant differences were found in implantation rates after exposure to a weak acid during the first cleavage of mouse embryos, despite detrimental effects on fetal weight and crown-rump length [170]. Additionally, evidence from a mouse model showed that culture in a suboptimal culture medium at 20% oxygen had a dramatic effect on gene expression related to energy production, cell survival, and cell development [171]. Generally, extensive manipulation and out of range in vitro conditions should be avoided when possible. These may not only impact embryo viability but also induce stress responses, triggering gene expression alteration or epigenetic modifications, which may affect adult health and the health of future generations [172].

- Cytoplasmic responses

Gametes are able to counteract OS mainly with enzymatic and non-enzymatic AOXs, which lie in their cytoplasm or extracellular fluids. Their levels can impact gamete quality and the chance of fertilization. Catalase, glutathione-S-transferase (GST), and arylesterase enzymes have been described in cumulus cells, with higher levels reported in responders to ART stimulation protocols. These patients showed low MDA levels and better oocyte quality compared to poor responders, which had a lower amount of AOX enzymes in cumulus cells [173]. Furthermore, exposure of human oocytes to growth hormone, physiologically secreted by granulosa cells, resulted in reduced oocyte apoptosis and OS markers in follicular fluid, and improved oocyte mitochondrial function and overall quality [174]. Recently, treatment with growth hormone resulted in increased oocyte fertilization and embryo formation in women with polycystic ovary syndrome [174].

Antioxidant systems in sperm include SOD, catalase, GPx, non-enzymatic factors with a low molecular weight such as glutathione, N-acetylcysteine, vitamins A, E, and C, coenzyme Q10, carnitines, myoinositol, and lycopene, and micronutrients such as selenium, zinc, and copper [175]. Mature sperm have low intracellular AOX levels due to the limited amount of cytoplasm. The deficiency of seminal AOX capacity exposes the sperm to OS-induced damage [176].

- Nuclear responses

High DNA compaction reduces the ability of nuclear responses to OS in sperm. In contrast, both oocytes and embryos are able to respond to OS through several AOX pathways. One of these include the nuclear factor-E2-related factor 2 (NRF2), a nuclear factor and a transcriptional activator of genes sharing the AOX response element in their promoters. The Kelch-like ECH-associated protein 1 (Keap1) counteracts the effect of NRF2 by contributing to its degradation by ubiquitin proteasomes [177]. In vitro exposure to isoniazid in mice oocytes led to an increase in ROS and to the activation of the OS-response pathway Keap1-NRF2 [178]. Similarly, in vitro exposure to OS resulted in an up-regulation of NRF2 expression and its downstream antioxidant genes in preimplantation bovine embryos at different times of development (8-cell, 16-cell, and blastocyst stage embryos). Furthermore, competent blastocysts were found having higher NRF2 expression and lower ROS levels, opposite to non-competent ones, which had lower NRF2 expression and higher ROS levels [179].

- Effects of out-of-range culture conditions on the gametes, embryos, and offspring

The best quality-controlled IVF laboratories avoid drastic excessive ROS insults to embryos and gametes coming from out-of-range culture conditions. This strategy may not be enough to completely scavenge excess ROS levels present in the culture medium compared to the ROS levels present in follicular fluid [123,180,181,182]. Excessive concentrations of ROS results in cell death, however, posed cells may exhibit an adaptive response to oxidation. The typical cell response is termed preconditioning [183]. However, ART itself has been proven to have a slight adverse effect on the offspring health. A recent cohort study performed on 764 children (382 conceived by ART and the rest 382 conceived naturally) found slightly higher blood pressure and changes in left ventricular structure and function in those conceived by ART compared to the controls [184]. Other investigators have reported an increased risk of autism spectrum disorders [185] and asthma, but not allergies [186] in children conceived from ART. 

When ROS are present at levels that allow gametes and embryos to develop into a live birth, there is a concern that early epigenetic modifications will produce DNA marks that will be expressed at the later stages of life and therefore have transgenerational effects [16,187]. A recent study evaluated the potential transgenerational inherited effects of environmental contaminants on the molecular alterations of sperm nuclear basic proteins (SNBP), DNA, and semen parameters of a family case, a father and son, living in the Land of Fires [188]. The authors of this study indicated high levels of seminal copper and chromium, alterations in SNBP content, and low DNA binding affinity of SNBPs. Interestingly, unstable DNA binding capacity was more evident in the son’s SNBP and was correlated with a lower seminal antioxidant activity. 

The human paternal genome is globally demethylated after fertilization, while the maternal genome is demethylated at a much slower rate in human embryos. Aberrant DNA methylation patterns are reported in ART patients as well as multiple imprinted loci, hypermethylation and hypomethylation events, and mosaic methylation errors, which are caused during the global demethylation after fertilization [189,190]. The tight association between ART and imprinting disorders was demonstrated by significant differences in the genome-wide DNA methylation observed in ART patients compared to spontaneously conceived patients [190]. It appears that most of the imprinting errors are produced at the zygote stage [180,181] because extending culture to the blastocyst stage does not appear to increase the risk for imprinting errors [191]. A study using abnormal in vitro culture conditions in mouse models indicated high expression of multiple growth-related imprinted genes, resulting in aberrant fetal growth and development and negative impact on the behavior of adult mice [192].

## 4. Strategies to Minimize OS in ART

General strategies to minimize OS rely on the reduction of ROS generation and/or enhancement of the AOX capacity. Herein, we highlight few strategies that may help control OS in an ART setting:

(a) Keeping optimal conditions of external factors

Laboratory design is highly important so that general conditions, such as room temperature, humidity, air pressure, and light, remain at optimal ranges. The location of the laboratory should be carefully chosen based on the assessment of environmental parameters to avoid areas with increased air pollution [193]. An air filtration system with high-efficiency particulate air and active carbon filters is necessary to improve air quality [91]. The number of incubators should be enough for the number of cases treated, depending on the type and capacity of the incubators [194]. Distances between incubators and workstations should be as short as possible to reduce the total time needed for each technique and avoid accidents [83].

Exposure of oocytes and embryos to suboptimal conditions in terms of temperature, pH, and oxygen is unavoidable, as many crucial steps of the IVF procedure, such as oocyte collection and denudation, ICSI, and embryo transfer, are performed outside the incubator. The pH can increase very quickly (in one or two minutes) after removal of the culture dish from the incubator [97,102]. These culture dishes, even if returned to the gassed incubator, will take a longer time (more than 30 min for non-oil-coated culture dishes, and about 35 min for droplets of 50 μL covered by oil) to re-equilibrate the pH after exposure to a non-gassed atmosphere [97,102]. Therefore, it is essential to keep the exposure of culture dishes to non-gassed atmospheres to a strict minimum [97,102]. Additionally, because of the design of disposable plastic dishes, there is no direct contact between the dish and the warming stage, and there is always an air gap formed that reduces the efficacy of the warming stages and allows the medium in the dish to cool down fast [54]. Therefore, time limitations should be imposed, and proper training of the laboratory staff is recommended to ensure faster handling and minimization of adverse events. Implementation of detailed standard operating procedures may allow keeping a limited number of oocytes in the pipette at a time and culturing a specific number of oocytes per dish.

ROS production in vitro may be caused by transient exposure of embryos to light [48]. Strategies to minimize the negative consequences of light include working under low illumination, applying red and green filters on the microscopes, and reducing the duration of oocyte and embryo handling. 

Quality control is essential to maintain all parameters in an acceptable range. Quality control devices, such as thermometers, pH meters, and VOC meters, should be calibrated and all results should be reported. According to the ESHRE guidelines, an alarm and monitoring system is necessary for all critical equipment, and this may help minimize the negative impact on embryo culture in case of unexpected events such as power failure [82].

(b) AOX supplementation in vitro
  i. AOX supplementation of culture media
    Early embryo development is not only reliant on the relevant nutrient supply, but is also crucially dependent on proper redox regulation in the different sections of the female genital tract [195]. Hence, attempts were made to reduce possible OS during the embryo culture by adding AOXs to the culture medium. A study reported significantly improved mitochondrial activity and blastocyst development in the AOX-supplemented group of aged female mice [196]. Furthermore, supplementation of culture medium with 1 nM of melatonin resulted in optimum oocyte development and nuclear maturation, whereas higher melatonin levels (105 to 107 nM) resulted in detrimental effects on nuclear maturation [197]. Additionally, a recent review highlighted the beneficial effects of AOX supplementation of culture media on the growth improvement of different species of embryos in vitro [198]. To the contrary, no significant effects on embryo development were observed following AOX supplementation of the culture medium [79].
    A recent prospective randomized multi-center study indicated the positive effect of supplementation of the embryo culture medium with acetyl-L-carnitine, N-acetyl-L-cysteine, and α-lipoic acid in increasing the implantation and pregnancy rates in women 35–40 years [199]. Interestingly, exposure to L-ascorbic acid (10 µM) and α-tocopherol (250 µM) has been shown to enhance the development of porcine denuded oocytes from metaphase I to metaphase II, and to protect against DNA fragmentation of cumulus cells [200].
    Due to the variation of oxygen tension in different sections of the female reproductive tract [70,75], the maintenance of appropriate redox levels at the relevant embryonic developmental stages is essential. Excessive amounts of AOXs have been shown to be detrimental to numerous organs and cell types, possibly through “reductive stress” [201,202,203,204] and also by paradoxically triggering mitochondrial ROS production and cytotoxicity via a glutathione mechanism [205]. Therefore, AOXs can damage cell proliferation and embryo development if not provided at the appropriate concentrations. Yet, although this concept has been shown to play a role in other cells and organs, it still must be proven in embryos.
    It has recently been shown that different culture media regularly used by ART laboratories have significantly different redox levels as measured by the MiOXSYS system [203]. Studies using mouse IVF indicated that lowering OS levels by adding AOXs to the embryo culture media are beneficial in maintaining embryo viability and development [206,207].
    Before a conclusion is made regarding the potential beneficial effects of in vitro AOX supplementation of embryo culture, a few points need to be noted. First, the oxygen tension to which the early embryo is exposed in the female genital tract, under physiologic conditions, is decreasing from fallopian tube to the uterus where the environment is almost anoxic [36]. Second, the embryo’s metabolism during this journey is dynamic [75]. Consequently, if the in vitro conditions in an embryo culture are not meeting the requirements in terms of the oxygen tension and the redox conditions for optimum development, the embryo development may be impaired or even arrested. Therefore, the development of new technologies that possibly address the changing conditions in the fallopian tubes, and clear understanding of the redox biology of the early embryo and its natural environment in the fallopian tube and the uterus may help improve the overall ART outcomes.
  ii. AOX supplementation of cryopreservation media
    In animals, nano-minerals, such as selenium and zinc, have been successfully added to the culture media of gametes or embryos, or used for cryopreservation protocols of spermatozoa, oocytes, and embryos, thereby achieving a better ART outcome [208]. The supplementation of the culture medium used for gametes or embryo with SOD, in cats, has shown its effectiveness in: (1) improving the blastocyst formation rate from low-quality oocytes [209], and (2) reducing cell apoptosis and improving survival of the cumulus and oocyte unit [210]. The addition of hypotaurine, during sperm preparation before ICSI, resulted in the reduction of SDF of frozen and thawed sperm [211]. In a recent meta-analysis, other AOXs were found to improve the motility and DNA integrity of cryopreserved gametes [212]. The latter study indicated that using AOXs in cryopreservation media could increase motility by 4.60% (95% CI 3.05–6.16), viability by 5.71% (95% CI 1.72–9.71), and DNA integrity by 10.20% (95% CI 7.42–12.98) in cryopreserved-thawed sperm.
    The addition of AOXs to vitrification media has been shown to decrease the OS level during the process and increase the embryo survival rate [213].

(c) AOX supplementation in vivo

The latest Cochrane systematic review on the use of AOXs for male subfertility collected data from 6264 patients referred to fertility clinics or undergoing ART [214]. The authors of the latter study indicated a significant improvement of live birth rate (14–26% vs. 12%) and clinical pregnancy rate (12–26% vs. 7%) compared to placebo, and no difference in miscarriage rate. However, the quality of the evidence was rated as low or very low due to the studies’ heterogeneity. Similarly, the latest Cochrane review on the use of AOXs for female subfertility showed a positive effect on live birth rate and clinical pregnancy rate [215]. Examining this study in detail, the authors found an improvement in live birth rates (OR 1.79, 95% CI 1.20 to 2.67, *p* = 0.005). However, this result was based on only 7 RCTs, for a total of 124 live births from 750 couples. The exclusion of studies with high risk of bias lead to the loss of the evidence of improvement (OR 1.38, 95% CI 0.89 to 2.16). Notably, the sample size and the number of events (live births) were further reduced (n = 540 and n = 105, respectively) and this may possibly explain the results, suggesting the need for further high-quality RCTs assessing this outcome. Finally, by including 11 RCTs, the authors reported a significant increase in clinical pregnancy rates (OR 2.97, 95% CI 1.91 to 4.63, *p* < 0.0001) compared to placebo or no treatment. However, the quality of evidence was rated as low or very low. Currently, there is no consensus on the use of AOXs in the in vitro ART procedures in humans.

(d) Hormone supplementation of culture media

Recently, preclinical data have drawn attention towards the potential benefits of the addition of hormones to embryo cultures. The addition of insulin-like growth factor 2 (usually present in follicular fluid) to the culture of aged oocyte from mice, prior to fertilization, resulted in improved oocyte competence and reduced ROS production, as well as the incidence of chromosome defects in the developing embryos [216].

## 5. Conclusions

Oxidative stress has been established as an essential factor in the pathogenesis of couples’ infertility and may have negative impact on the outcomes of ARTs. In this comprehensive review, we provided the details of endogenous and exogenous sources of OS in an ART setting. These may include gametes and embryos, as well as several external factors. In addition, we discussed the potential negative effects of OS on gametes and embryos. Although gametes and embryos are partially able to counterbalance OS, this may result in permanent and inheritable epigenetic effects, cell death, and poor reproductive outcomes. We have also provided strategies to reduce the production of ROS in the ART laboratory and to manage OS during the entire human embryo culture period, from gametes to blastocyst stage. Implementation of these strategies by ART practitioners may help optimize embryo culture conditions, minimize the impact of OS on gametes and embryos, and improve the overall ART outcomes.

## Abbreviations

AOXs: antioxidants; ARTs: assisted reproductive techniques; ATP: adenosine triphosphate; GPx: glutathione peroxidase; GST: glutathione-S-transferase; H_2_O_2_: hydrogen peroxide; HEPA: high-efficiency particulate air; HVAC: heating ventilation and air conditioning; ICSI: intracytoplasmic sperm injection; IUI: intrauterine insemination; IVF: in vitro fertilization; Keap1: Kelch-like ECH-associated protein 1; MDA: malondialdehyde; MMP: mitochondrial membrane potential; NO: nitric oxide; NRF2: nuclear factor-E2-related factor 2; O_2_^•−^: superoxide anion; ^•^OH: hydroxyl radicals; OS: oxidative stress; pHe: external pH; pHi: internal pH; Ppb: parts per billion; pO_2_: atmospheric oxygen; POV: peroxide value; PUFA: polyunsaturated fatty acids; ROS: reactive oxygen species; SDF: sperm DNA fragmentation; SOD: superoxide dismutase; VOCs: volatile organic compounds
